# Vaginal Microbiome Composition in Early Pregnancy and Risk of Spontaneous Preterm and Early Term Birth Among African American Women

**DOI:** 10.3389/fcimb.2021.641005

**Published:** 2021-04-29

**Authors:** Anne L. Dunlop, Glen A. Satten, Yi-Juan Hu, Anna K. Knight, Cherie C. Hill, Michelle L. Wright, Alicia K. Smith, Timothy D. Read, Bradley D. Pearce, Elizabeth J. Corwin

**Affiliations:** ^1^ Emory University Nell Hodgson Woodruff School of Nursing, Atlanta, GA, United States; ^2^ Department of Family & Preventive Medicine, Emory University School of Medicine, Atlanta, GA, United States; ^3^ Department of Gynecology and Obstetrics, Emory University School of Medicine, Atlanta, GA, United States; ^4^ Department of Biostatistics and Bioinformatics, Emory University Rollins School of Public Health, Atlanta, GA, United States; ^5^ School of Nursing, University of Texas at Austin, Austin, TX, United States; ^6^ Division of Infectious Diseases, Department of Medicine, Emory University School of Medicine, Atlanta, GA, United States; ^7^ Department of Epidemiology, Emory University Rollins School of Public Health, Atlanta, GA, United States; ^8^ Columbia University School of Nursing, New York, NY, United States

**Keywords:** microbiome, microbiota, pregnancy, preterm birth, early term birth, gestational age at birth

## Abstract

**Objective:**

To evaluate the association between the early pregnancy vaginal microbiome and spontaneous preterm birth (sPTB) and early term birth (sETB) among African American women.

**Methods:**

Vaginal samples collected in early pregnancy (8-14 weeks’ gestation) from 436 women enrolled in the Emory University African American Vaginal, Oral, and Gut Microbiome in Pregnancy Study underwent 16S rRNA gene sequencing of the V3-V4 region, taxonomic classification, and community state type (CST) assignment. We compared vaginal CST and abundance of taxa for women whose pregnancy ended in sPTB (N = 44) or sETB (N= 84) to those who delivered full term (N = 231).

**Results:**

Nearly half of the women had a vaginal microbiome classified as CST IV (Diverse CST), while one-third had CST III (*L. iners* dominated) and just 16% had CST I, II, or V (non-iners *Lactobacillus* dominated). Compared to vaginal CST I, II, or V (non-iners *Lactobacillus* dominated), both CST III (*L. iners* dominated) and CST IV (Diverse) were associated with sPTB with an adjusted odds ratio (95% confidence interval) of 4.1 (1.1, infinity) and 7.7 (2.2, infinity), respectively, in multivariate logistic regression. In contrast, no vaginal CST was associated with sETB. The linear decomposition model (LDM) based on amplicon sequence variant (ASV) relative abundance found a significant overall effect of the vaginal microbiome on sPTB (p=0.034) but not sETB (p=0.320), whereas the LDM based on presence/absence of ASV found no overall effect on sPTB (p=0.328) but a significant effect on sETB (p=0.030). In testing for ASV-specific effects, the LDM found that no ASV was significantly associated with sPTB considering either relative abundance or presence/absence data after controlling for multiple comparisons (FDR 10%), although in marginal analysis the relative abundance of *Gardnerella vaginalis* (p=0.011), non-iners *Lactobacillus* (p=0.016), and *Mobiluncus curtisii* (p=0.035) and the presence of *Atopobium vaginae* (p=0.049), BVAB2 (p=0.024), *Dialister microaerophilis* (p=0.011), and *Prevotella amnii* (p=0.044) were associated with sPTB. The LDM identified the higher abundance of 7 ASVs and the presence of 13 ASVs, all commonly residents of the gut, as associated with sETB at FDR < 10%.

**Conclusions:**

In this cohort of African American women, an early pregnancy vaginal CST III or IV was associated with an increased risk of sPTB but not sETB. The relative abundance and presence of distinct taxa within the early pregnancy vaginal microbiome was associated with either sPTB or sETB.

## Introduction

Nearly 1 in 10 US infants are born preterm (< 37 weeks gestation) (Martin et al., 2019), making preterm birth (PTB) a leading cause of infant morbidity and mortality (Callaghan et al., 2006; Mathews and Driscoll, 2017). African American (AA) women experience a PTB rate 1.5 times that of white women (Martin et al., 2019). While low socioeconomic status is a risk factor, less than half of the US black-white disparity in PTB is explained by socioeconomic status and other known risk factors (McGrady et al., 1992; Goldenberg et al., 2008; Kramer and Hogue, 2009). The Institute of Medicine has called for research aimed at understanding factors that contribute to the high US rates of PTB, particularly among AA women, as crucial for reducing US infant morbidity and mortality (Behrman and Butler, 2007).

A growing body of research has focused upon the prenatal vaginal microbiome in shaping risk for PTB. Most 16S rRNA gene sequencing surveys characterize the vaginal microbiome using a set of community state types (CST) that were defined *via* hierarchical clustering and consideration of predominant taxa, with communities clustered into five CST: four dominated by *Lactobacillus* spp., includin*g L. crispatus* (CST I), *L. gasseri* (CST II), *L. iners* (CST III), or *L. jensenii* (CST V), and a fifth with a lower proportion of lactic acid producing bacteria and higher proportion of anaerobes (CST IV) (Zhou et al., 2007; Ravel et al., 2011). A consistent finding is that the proportion of women classified into a specific CST varies by race, with AA women significantly more likely to have a diverse vaginal CST not dominated by *Lactobacillus* (Zhou et al., 2007; Ravel et al., 2011; MacIntyre et al., 2015). Among women whose vaginal microbiome is dominated by *Lactobacillus*, the predominant species also varies by race, with *L. crispatus* more commonly predominating among white women and *L. iners*, more commonly predominating among AA women (Hyman et al., 2014). Because *L. iners* produces less acid than other *Lactobacillus* spp., it is less effective at maintaining the low pH that typically characterizes vaginal health (Amabebe and Anumba, 2018). AA women are also significantly more likely than women of other races/ethnicities to have a vaginal microbiome that harbors *Gardnerella*, BV-associated bacterium-1 (BVAB1, candidate name *Candidatus Lachnocurva vaginae*), and other pathogenic bacteria associated with invasion of the amniotic cavity (Fettweis et al., 2014). Social, environmental, and behavioral exposures driving racial/ethnic differences in the vaginal microbiome remain to be explored, although factors associated with life course exposures to stress and adversity as well as socioeconomic status are likely to contribute (Cammack et al., 2011; Dunlop et al., 2019 *).*

To date, studies of the vaginal microbiome and PTB have yielded somewhat conflicting findings both within and across racial/ethnic groups. To illustrate, lower *Lactobacillus* and higher *Gardnerella* abundance have been associated with PTB (DiGiulio et al., 2015) and preterm premature rupture of membranes (Brown et al., 2016) in two mostly white cohorts, whereas no such associations were found in mostly AA cohorts (Romero et al., 2014a; Nelson et al., 2016; Stout et al., 2017). A cohort of mostly white and Asian women with prior spontaneous PTB (sPTB) also found no association between diverse vaginal communities and PTB, but did identify that higher abundance of *L. iners* increased and *L. crispatus* decreased subsequent PTB risk (Kindinger et al., 2017). Application of a refined bioinformatics approach that improved *Lactobacillus* species classification found no association between *L. iners* and birth outcome in either the white or AA cohorts, a protective effect of *L. crispatus* in both cohorts, and an association between lower abundance of *L. gasseri* and *L. jensenii* and PTB only in the AA cohort (Callahan et al., 2017b). In a multi-race cohort, the presence of *Mycoplasma* was found to increase PTB risk and the presence of *Mageebacillus indoclicus* (previously referred to as BVAB3) to drastically decrease PTB risk for minority women, while no associations were observed for white women (Wen et al., 2013).

The discordance in findings across studies may reflect differences in study sample. Across studies, there is considerable variation in participant racial/ethnic and socioeconomic diversity, characteristics themselves that are linked with PTB (Dunlop et al., 2019), and many studies are small in size or had a limited number of AA women. In addition, across studies there is substantial heterogeneity in the definition and classification of PTB, with many not distinguishing sPTB from any PTB, including those medically-indicated due to maternal or fetal complications. The clinical factors preceding PTB types vary; the spontaneous type is more often linked with intra-uterine infection or inflammation, whereas the indicated type is often related to medical complications (Behrman and Butler, 2007). There is also heterogeneity in classification of the term birth comparison group. Across microbiome-PTB studies, many classify women whose births fall into the early term category (37^0/7^ through 38^6/7^ weeks) as term controls, whereas others use 39 weeks and greater, while some do not clearly define the gestational age limits of their comparison group. To date, no vaginal microbiome studies have specifically differentiated early term birth from full term birth despite recommendations to address risk factors for early term birth, as infants born early term experience excess morbidity relative to infants born full term (between 39^0/7^ and 40^6/7^ weeks) (American College of Obstetricians and Gynecologists, 2013). Finally, there is also considerable variability in the gestational age of vaginal sample collection across studies, and it is established that the composition of the vaginal microbiome changes with advancing gestational age (Romero et al., 2014a; Romero et al., 2014b) becoming more stable and less likely to shift from a diverse CST to one dominated by *Lactobacillus* spp.

The main objective of this study was to characterize the composition of the vaginal microbiome in early pregnancy (8-14 weeks’ gestation) among a cohort of AA women and evaluate its association with the occurrence of sPTB and sETB in comparison to full term birth. We restricted the study to AA women based on a health disparity research framework that recommends as a first step to understanding health disparities to look within the high burden group to identify intra-group risk and protective factors. (Rowley et al., 1993) This is especially pertinent given that existing studies of the vaginal microbiome in pregnancy often do not consider socioeconomic status and hence are not able to parse the possibly competing or additive effects of race and socioeconomic status.

## Methods

### Participants

Participants for this study were from the Emory University African American Vaginal, Oral, and Gut Microbiome in Pregnancy Study (Corwin et al., 2017). African American women were recruited from prenatal clinics of two hospitals in Atlanta, GA: a private hospital that provides services for a socioeconomically diverse group of women and a public facility that primarily provides services for low-income women. Inclusion criteria for enrollment into the cohort included: 1) being African American, defined as being of self-reported Black/African American race and born in the United States; 2) presenting with a singleton pregnancy between 8-14 weeks gestation (verified by prenatal record); 3) ability to comprehend written and spoken English; 4) age between 18-40 years; and 5) absence of the diagnosis of any chronic medical condition or the chronic use of prescription medication to manage a health condition (verified by prenatal record) in order to reduce the number of early deliveries attributed to medical indications and focus on spontaneous early births. Women who developed health conditions or pregnancy complications, including those who required prescription medications were retained in the study and these exposures and their gestational age of occurrence were recorded. Participants in the present study included the first consecutively enrolled 474 women whose pregnancy ended in a birth for whom at least one vaginal swab sample from the enrollment study visit was available for DNA extraction and 16S rRNA gene sequencing. The research protocol was reviewed and approved by the Emory University Institutional Review Board (protocol number 68441); all participants provided written informed consent.

### Data Collection

The collection of biological samples, questionnaire and clinical at two points during pregnancy (at prenatal care visits occurring between 8-14 and 24-30 weeks’ gestation), and clinical data (from the medical record) post-delivery has been described in detail previously (Corwin et al., 2017). Items relevant to this study are summarized below.

#### Questionnaire Data

Sociodemographic survey based on maternal self-report and prenatal administrative record review was used to ascertain maternal age upon entry into the study, years of education (less than high school, high school or GED, some college, college graduate), and prenatal health insurance type (categorized as low-income Medicaid, Right-from-the-Start Medicaid, private insurance). In Georgia, to be eligible for low-income Medicaid during pregnancy a woman must have a household income at or below 133 percent of the federal poverty level, whereas to be eligible for Right-from-the-Start Medicaid coverage during pregnancy, women must have a household income at or below of 200 percent of the federal poverty level (Georgia Medicaid, 2020).

A health survey was used to ascertain substance use (alcohol, tobacco, marijuana, other drugs) within the month prior to the visit the use of substances. For each item for which there is occurrence, the timeline follow back approach (Sobell and Sobell, 1996; Carey et al., 2001; Robinson et al., 2014); was used to ascertain the timing and frequency of occurrence. These data were used to classify women as users of alcohol, tobacco, and/or marijuana in the month prior to the enrollment visit.

#### Medical Record Data

Medical chart abstraction was completed by the research team using a standardized chart abstraction tool to ascertain the following characteristics, conditions and birth outcomes: (1) Parity, categorized according to whether the woman had any prior birth or not and if prior births were term or preterm; (2) First prenatal body mass index (BMI), calculated from measured height and weight at the first prenatal visit between 8-14 weeks gestation and categorized according to accepted definitions (obesity ≥ 30 kg/m^2^, overweight 25-29.99 kg/m^2^, healthy weight 18.5-24.99 kg/m^2^, and underweight <18.5 kg/m^2^); (3) Genitourinary tract infections and antibiotic use were noted based on physician diagnosis and/or laboratory results in the record for bacterial vaginosis, chlamydia, gonorrhea, trichomoniasis, or urinary tract infection and any prescription for antibiotics; the gestational age of diagnosis of infections or prescription of antibiotics were ascertained by comparing the date of these occurrences to the estimated date of confinement based on the last menstrual period (LMP) and/or ultrasound before 14 weeks’ gestation according to standard clinical criteria (American College of Obstetricians and Gynecologists, 2014); participants were coded as having been exposed to infection or antibiotics in the month prior to the enrollment visit if they had an infection or were prescribed an antibiotic in the four weeks prior; (4) Gestational age at birth was determined from the delivery record using the best obstetrical estimate (American College of Obstetricians and Gynecologists, 2014) based upon the date of delivery in relation to the estimated date of confinement established by the 8-14 week prenatal visit. All participants received early pregnancy dating by last menstrual period (LMP) and/or early ultrasound, given enrollment criteria. (5) Type of Labor (spontaneous, induced, none) and mode of delivery (vaginal, C-section) along with indication for induction and/or C-section were obtained and used to phenotype birth outcomes.

Pregnancy outcomes were classified into the following outcome categories: Preterm birth (between 22^0/7^ and 36^6/7^ weeks gestation) further classified as spontaneous (following spontaneous labor or premature ruptures of membranes) or medically-indicated (following C-section or induction for an indication) (Ananth and Vintzileos, 2008); early term birth (between 37^0/7^ and 38^6/7^ weeks gestation) further classified as spontaneous or medically-indicated; and full term birth (39^0/7^ weeks or greater).

#### Vaginal Samples

Participants were provided verbal and pictorial instructions explaining how to obtain self-collected vaginal swabs. Vaginal microbiome sampling involved sampling the midportion of the vaginal vault (3-4 inches from introitus). Consistent with the protocols of the Human Microbiome Project (McInnes and Cutting, 2010), the sampling used a Sterile Catch-All™ Sample Collection Swab (Epicentre Biotechnologies, Madison WI) that was immediately handed to the study coordinator for placement in MoBio bead tubes (MoBio Laboratories, Inc.), that were frozen upright on ice until transported to the lab, where they were stored at −80°C until DNA extraction. Studies support that vaginal self-collection swabs sample the same microbial diversity as physician-collected swabs of the mid-vagina and have high overall morphotype-specific validity compared with provider-collected swabs (Forney et al., 2010). Additional self-collected vaginal swabs were obtained for pH (measured *via* pH-strips [Merck, Darmstadt, Germany] with a scale from 4.0-7.7), Nugent scoring *via* Gram staining, and measurement of vaginal cytokines. Gram staining was performed at the Emory Clinical Microbiology Laboratory, with smeared slides dried and heat-fixed prior to Gram staining and the slide scored *via* Nugent’s criteria (scores 0-3 are categorized as normal, 4-6 as intermediate, and 7-10 as bacterial vaginosis) (Nugent et al., 1991). Vaginal swabs were analyzed for cytokines, including interferon-γ, interleukin (IL)-6, IL-8, IL-10, and TNF-α, using the MesoScale assay platform (MesoScale Diagnostics Rockville, Maryland), which uses electrochemiluminescence for high sensitivity and broad dynamic range, according to manufacturer protocols. Vaginal swabs were also analyzed for C-reactive protein using enzyme-linked immunosorbent assay (ELISA) kits (R&D systems, cat# SCRP00) following the manufacture protocol. Vaginal fluid was used undiluted after centrifugation. The concentration of C-reactive protein was calculated based on the four parameter calibration curves generated for each set of samples assayed using the BioTek Gen5 software.

### DNA Extraction, Library preparation, Sequencing, and Bioinformatic Processing

DNA was extracted from participant swab samples using the DNeasy PowerSoil Kit (cat# 12888-100, Qiagen). DNA quantification based on a threshold of 5 ug/nL was used to identify samples that were borderline in terms of DNA yield; in cases that were borderline, DNA quality was assessed on a 2% agarose gel and quantitated with the Broad Range Quant-It kit from ThermoFisher Scientific (Q33130). Participant samples with DNA visible on the gel were sequenced as were 30 no-template controls (that contained all assay components except for DNA to verify lack of contamination across reagents and samples) and positive controls (that were a mixture of 20 vaginal specimens of known composition). Microbial composition was characterized by DNA sequencing of the 16S rRNA gene.

Amplification of the V3–V4 regions of the 16S rRNA gene was performed using a two step-PCR described previously (Elovitz et al., 2019). Briefly, the first PCR used the short 16S rRNA gene specific primers 319F (ACACTGACGACATGGTTCTACA[0–7]ACTCCTRCGGGAGGCAGCAG) and 806R (TACGGTAGCAGAGACTTGGTCT[0–7]GGACTACHVGGGTWTCTAAT) where the underlined sequence is the Illumina sequencing primer sequence and [0–7] indicate the presence of an heterogeneous pad sequence to improve sequencing quality (Fadrosh et al., 2014), for a total of 20 cycles. The second step extends the amplicon with the Illumina required adaptor sequences and the sample specific dual barcode system *via* 10 cycles with primers H1 (AATGATACGGCGACCACCGAGATCTACACNNNNNNNNACACTGACGACATGGTTCTACA) and H2 (CAAGCAGAAGACGGCATACGAGATNNNNNNNNTACGGTAGCAGAGACTTGGTCT) where NNNNNN indicates a sample specific barcode sequence and the underlined sequence corresponds to the Illumina sequencing primer for priming to the first step amplicon (Fadrosh et al., 2014). Amplicons were visualized on a 2% agarose gel, quantified, pooled in equimolar concentration, and purified prior to sequencing on an Illumina HiSeq 2500 (San Diego, CA, USA) modified to generate 300 bp paired-end reads (Holm et al., 2019). Extraction and PCR negative controls as well as a positive control composed of a mixture of 20 vaginal biological specimens of known composition were processed in parallel.

The sequences were de-multiplexed using the dual-barcode strategy, a mapping file linking barcode to samples and split_libraries.py, a QIIME-dependent script (Kuczynski et al., 2012). The resulting forward and reverse fastq files were split by sample using the QIIME-dependent script split_sequence_file_on_sample_ids.py, and primer sequences were removed using TagCleaner (version 0.16) (Schmieder et al., 2010). Further processing followed the DADA2 Workflow for Big Data and dada2 (v. 1.5.2) (https://benjjneb.github.io/dada2/bigdata.html) (Callahan et al., 2016). Forward and reverse reads were each trimmed using lengths of 255 and 225 bp, respectively, and were filtered to contain no ambiguous bases, have minimum quality score of 2, and were required to contain less than two expected errors based on their quality score. The relationship between quality scores and error rates were estimated for both sequencing runs to reduce batch effects arising from run-to-run variability. Reads were assembled and chimeras removed as per dada2 protocol.

Taxonomy was assigned to each amplicon sequence variant (ASV) generated by dada2 using PECAN (version 1.0), a rapid per sequence classifier (http://ravel-lab.org/speciateit), which classifies 16S rRNA gene sequences using Markov Chain models built from a curated set of reference sequences. Tables including total sequence counts for or relative abundances of 666 taxa for each sample with more than 2000 sequences were generated and used to calculate Shannon (richness) and Chao1 measures of alpha-diversity and rarefaction curves. A community state type (CST) was assigned to each sample using hierarchical clustering with the Jensen-Shannon divergence and Ward linkage (Ravel et al., 2011; Gajer et al., 2012). CST I is predominated by *L. crispatus*, CST II by *L. gasseri*, CST III by *L. iners*, CST IV was defined as lacking *Lactobacillus* predominance and comprising a diverse set of strict and facultative anaerobes, further split into CST IV-A (predominated by BVAB and *Gardnerella*), IV-B (predominated by *Atopobium* and *Gardnerella*), and IV-D (only anaerobes), while CST V is predominated by *L. jensenii*.

For association analyses, all taxa that were not assigned at the genus level were removed. Read counts for ASVs assigned to the same taxonomy (i.e., to a taxon defined by species and genus when species was available, or to a taxon defined only by genus when species was not available). All non-iners *Lactobacilli* were pooled; we assumed that any lactobacilli that could not be assigned at the genus level would not be *L. iners*, since it is well characterized. We further removed taxa found in fewer than 5 samples of the combined data from the 44 women who experienced sPTB and the 231 women who delivered full term. This trimming was conducted in two steps; first, taxa defined by genus *and* species were combined with the corresponding genus-only taxon if they occurred in fewer than 5 samples; then, genus-only taxa were removed if they occurred in fewer than 5 samples.

### Statistical Analyses

We calculated the proportion of participating women whose early pregnancy vaginal sample (collected during the enrollment visit at 8-14 weeks gestation) was categorized into each CST. Due to the low proportion of samples assigned to CST I, II, and V, these CST were combined into a single category creating three CST categories: non-iners *Lactobacillus* dominated (CST I, II, V), *Lactobacillus iners* dominated (CST III), non-*Lactobacillus* dominated or Diverse (CST IV). We summarized characteristics of samples as well as sociodemographic and clinical characteristics of women according to CST categories and compared differences in characteristics using Chi-square or t-test, as appropriate. We also summarized sociodemographic and clinical characteristics of women according to birth outcome categories and compared differences in characteristics using Chi-square or t-test, as appropriate.

We tabulated the proportion of women whose early pregnancy vaginal sample was categorized into each CST and CST category and compared the proportion of women with sPTB and sETB (in separate models) vs. the proportion of women with a full term birth across the CST categories using the Chi-square test. We also displayed the relative abundance of the 25 taxa of highest relative abundance in the vaginal samples by CST and gestational age categories *via* a heat map. To evaluate the association between vaginal CST (I through IV) and CST category (non-iners *Lactobacillus*, *Lactobacillus iners*, or Diverse) in early pregnancy and gestational age at birth outcomes, we then performed Firth-corrected logistic regression, contrasting sPTB and sETB with full term birth in separate models, according to vaginal CST category (with non-iners *Lactobacillus* as the referent category). We used the Firth correction for the relatively rare occurrence of the gestational age outcomes of interest (especially sPTB) among those with non-iners *Lactobacillus* CST, which reduces small sample bias in maximum likelihood estimation (King and Zeng, 2001). In addition to CST, all logistic regression models included the following co-variates, selected on the basis of their association with gestational age at birth and vaginal microbiome composition based on the existing literature (Behrman and Butler, 2007; Dunlop et al., 2019) as well as bivariate associations within this cohort (**Tables 3**, **4**): maternal age, level of education, insurance type, marital-cohabitation status, parity, first prenatal BMI, tobacco use, marijuana use, and gestational age at sample acquisition. In some cases, the upper confidence limits for the odds of sPTB were very large and appeared to support a directional hypothesis; in these cases, we also give one-sided intervals at the same confidence level (Ruxton and Neuhäuser, 2010).

To test for both the global effect of the vaginal microbiome on the outcomes of interest as well as ASV-specific effects we used the linear decomposition model (LDM). The LDM allows for complex fixed-effects models, such as those that include multiple variables of interest whether continuous or categorical, their interactions, as well as other co-variates. Furthermore, it is permutation-based and, as such, can accommodate clustered data and maintain validity for small sample sizes and when data are subject to over-dispersion (Hu and Satten, 2020). The LDM analyses likewise adjusted for maternal age, level of education, insurance type, marital-cohabitation status, parity, first prenatal BMI, tobacco use, marijuana use, and gestational age at sample acquisition. The LDM provides an overall (global) test of association between the microbiome and traits of interest such as PTB, as well as a list of taxa that are individually associated with the trait that controls false discovery rate (FDR) at a pre-specified level; in these analyses, we set the FDR at 10%. We performed LDM analyses using relative abundance data as well as using a binary variable indicating presence or absence of each taxon. Analyses based on presence-absence were rarefied to the minimum library size, and averaged over rarefactions (Hu and Satten, 2021). To test for the effect of particular taxa that have previously been reported as associated with preterm birth on the occurrence of sPTB in our cohort, we also report *p*-values from the LDM based on relative abundances and presence/absence of the following 19 taxa: *Aerococcus christensenii, Atopobium vaginae, BVAB1* (candidate name *Candidatus Lachnocurva vaginae*), BVAB2, *Dialister microaerophilus, Finegoldia magna, Gardnerella vaginalis, Lactobacillus iners*, non*-iners Lactobacillus, Megasphaera, Mobiluncus curtisii, Mycoplasma hominis, Prevotella amnii, Prevotella bivia, Prevotella buccalis, Prevotella timonensis, Ureaplasma urealyticum, Sneathia amnii, and Sneathia sanguinegens*.

## Results

### 16S rRNA Gene Sequencing Data

Among the 474 cohort participants with vaginal samples from early pregnancy (8-14 weeks gestation) that were included in this study, 38 samples were removed due to low library size (fewer than 2,000 reads). A rarefaction curve displaying rarefaction depth by richness is given in **Supplement Figure 1**. From the remaining 436 samples, a total of 20,097,432 reads were grouped into 666 ASVs. After removing taxa that were only identified to the family level or higher, we retained 19,571,748 reads in 601 ASVs. After the glomming and trimming operation, the data remaining comprised 19,544,087 reads in 324 taxa (with a range of 2,978 to 181,072 reads per sample). For samples run in duplicate (N=7), the sample with the higher read count was retained and the other dropped before creation of the biome table.

### Sample and Participant Characteristics According to CST

**Table 1** shows the distribution of vaginal CST at the early pregnancy enrollment visit (8-14 weeks gestation) among the 436 study participants. Nearly half of the women had a vaginal microbiome classified as CST IV (Diverse CST), with CST IV-A (26.8%) and CST IV-B (21.1%) being substantially more common than CST IV-C (1.1%). Approximately one-third had CST III (*L. iners* dominated), while just 16% classified as CST I, II, or V, with most of these being dominated by *L. crispatus* (11.5%) compared to *L. gasseri* (1.8%) and *L. jenseni* (3%). Because of the small proportion of women with CST I, II, and V, we combined these into a single CST category for statistical analyses (referred to as non-iners *Lactobacillus* CST).

**Table 1:** Distribution of Vaginal Microbiome Community State Type among Study Participants.

CST	Characteristic Taxa	No. (%) of Cohort N = 436
I	*Lactobacillus crispatus*	50 (11.5%)
II	*Lactobacillus gasseri*	8 (1.8%)
III	*Lactobacillus iners*	151 (34.6%)
IV	Diverse	214 (49.1%)
*IV-A*	*BVAB, Gardnerella*	*117 (26.8%)*
*IV-B*	*Atopobium, Gardnerella*	*92 (21.1%)*
*IV-C*	*Anaerobes^1^*	*5 (1.1%)*
V	*Lactobacillus jenseni*	13 (3.0%)

^1^Common anaerobes include Aerococcus, Dialister, Eggerthella, Finegoldia, Megasphaera, Mobiluncus, and Prevotella.

**Figure 1:**
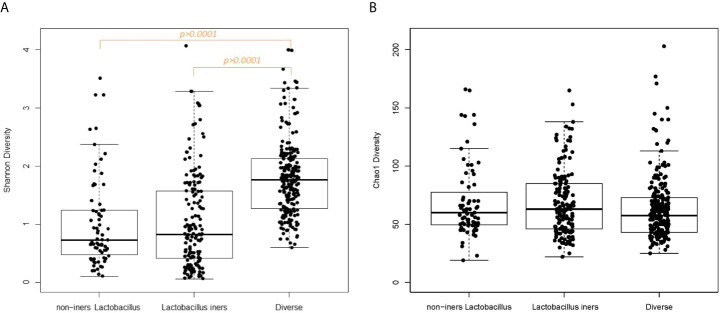
**(A)** Box plot Shannon diversity according to CST category. **(B)** Box plot of Chao1 diversity according to CST category.

In examining vaginal sample characteristics by CST category, there were significant differences in sample read count, pH, Nugent score, Shannon diversity and the log_10_-transformed vaginal concentrations of C-reactive protein and cytokines index, but no significant difference in vaginal white blood cell quantification or Chao1 diversity (**Table 2A**). Box plots of the alpha-diversity measures across the CST categories demonstrate that the Diverse CST, which had the smaller library size, had significantly higher Shannon index compared to the non-iners *Lactobacillus* CST and the *Lactobacillus iners* CST (**Figure 1A**, p<0.0001 for both comparisons). The combination of similar Chao1 diversity across CSTs (**Figure 1B**) but higher Shannon diversity (evenness) moving from the non-iners *Lactobacillus* CST to *Lactobacillus iners* CST to the Diverse CST suggests the main feature differentiating the CST categories is the increasing population of the rare taxa along this axis, rather than in increase in the number of taxa.

**Table 2a:** Early Pregnancy Vaginal Sample Characteristics According to CST Category.

CHARACTERISTIC	Non-iners *Lactobacillus *(CST I, II, V) N = 71	*Lactobacillus iners *(CST III) N = 151	Diverse (CST IV) N = 214	p-value^1^
Gestational weeks, mean ± sd	11.5 ± 1.9	11.47 ± 2.7	11.0 ± 2.4	0.158
Read count, mean ± sd	50,053 ± 27,826	52,327 ± 26,624	40,385 ± 19,724	***0.0001***
pH, mean ± sd	4.61 ± 0.37	4.59 ± 0.33	4.75 ± 0.43	***0.001***
Nugent score, mean ± sd	1.75 ± 2.29	2.85 ± 2.67	7.19 ± 2.49	***0.0001***
`Nugent score				
Normal	57 (84%)	93 (65%)	20 (10%)	***0.0001***
Intermediate	5 (7%)	31 (22%)	33 (16%)	
Bacterial vaginosis	6 (9%)	20 (14%)	155 (75%)	
White blood cells				
None	40 (59%)	66 (46%)	102 (49%)	0.130
Rare	12 (18%)	41 (29%)	54 (26%)	
Few	10 (15%)	34 (24%)	44 (21%)	
Moderate	5 (7%)	3 (2%)	5 (2%)	
Many	1 (2%)	0 (0%)	3 (1%)	
Chao1 diversity	69.37 ± 31.98	68.57 ± 28.25	63.58 ± 27.59	0.159
Shannon diversity	1.02 ± 0.78	1.04 ± 0.80	1.79 ± 0.65	***0.0001***
Cytokines (log_10_, mg/dL)				
Interferon-γ	-0.89 ± 0.23	- 0.88 ± 0.20	- 0.30 ± 0.17	***0.043***
Interleukin-6	-0.85 ± 0.27	- 0.22 ± 0.18	0.51 ± 0.15	***0.0001***
Interleukin-10	-3.48 ± 0.25	-3.69 ± 0.20	-2.96 ± 0.14	***0.005***
TNF-α	-2.54 ± 0.26	- 2.19 ± 0.18	-0.89 ± 0.13	***0.0001***
C-reactive protein (log_10_, mg/dL)	-10.6 ± 0.19	-9.8 ± 0.16	-8.7 ± 0.12	***0.0001***

^1^p-value for t-test (continuous variables) or Fisher’s exact or Chi-square test (categorical variables); bold indicates statistical significance for α = 0.05.

**Figure 2:**
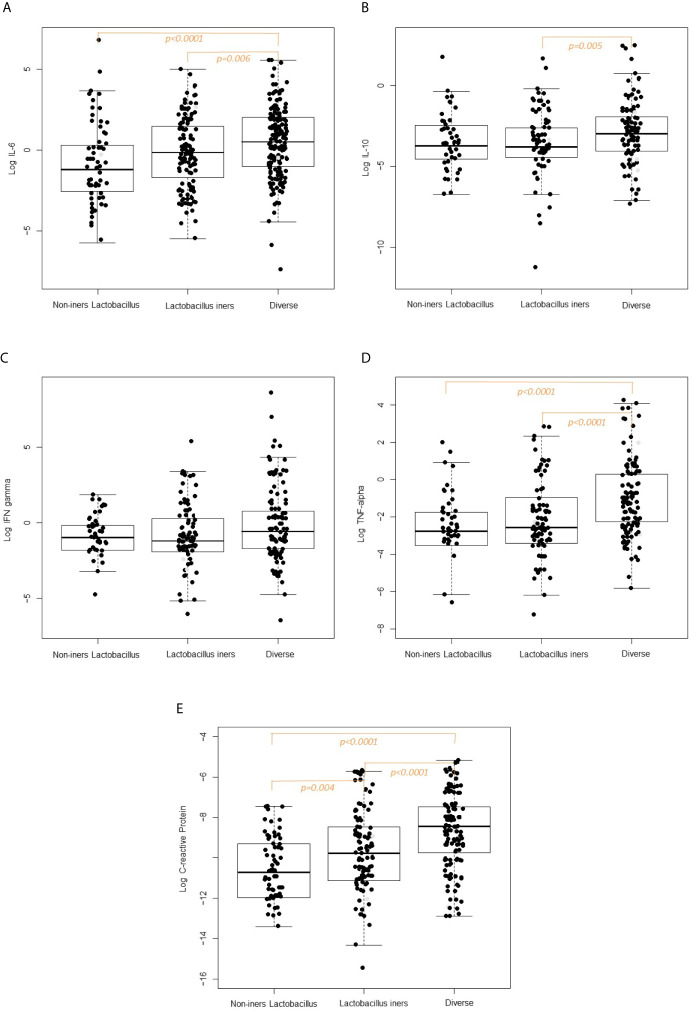
**(A)** Vaginal Interleukin-6 concentration according to CST category. **(B)** Vaginal Interleukin-10 concentration according to CST category. **(C)** Vaginal Interferon-gamma concentration according to CST category. **(D)** Vaginal TFN-alpha concentration according to CST category. **(E)** Vaginal C-reactive concentration according to CST category.

Box plots of the log_10_-transformed vaginal concentrations of C-reactive protein and cytokines across the CST categories are shown in **Figure 2** and the pairwise differences in log_10_-transformed vaginal concentrations of C-reactive protein and cytokines by CST category are given in **Figure 2** and **Table 2B**. When comparing the non-iners *Lactobacillus* to the *Lactobacillus iners* CST category, there was a pairwise significant difference in the concentration of C-reactive protein (p=0.004) but not the vaginal cytokines. When comparing the non-iners *Lactobacillus* CST to the Diverse CST, there were significant differences in vaginal concentrations of IL-6 (p<0.0001), TNF-α (p=p<0.0001) and C-reactive protein (p<0.0001). When comparing the *Lactobacillus iners* CST to the Diverse CST, there were significant differences in vaginal concentrations of IL-6 (p=0.006), IL-10 (p=0.005), TNF-α (p<0.0001) and C-reactive protein p<0.0001).

**Table 2b:** Vaginal C-reactive protein and Cytokine Concentrations According to CST Category.

Vaginal Measure	Non-iners *Lactobacillus *(CST I, II, V) N = 71	*Lactobacillusiners *(CST III) N = 151	Diverse (CST IV) N = 214
	**p-value*^1^***		
Interferon-γ			
Non-iners Lactobacillus	–	1.0	0.256
Lactobacillus iners	1.0	–	0.073
Diverse	0.256	0.073	–
Interleukin-6			
Non-iners Lactobacillus	–	0.142	***0.0001***
Lactobacillus iners	0.142	–	***0.006***
Diverse	***0.0001***	***0.006***	–
Interleukin-10			
Non-iners Lactobacillus	–	1.0	0.239
Lactobacillus iners	1.0	–	***0.005***
Diverse	0.239	***0.005***	–
TNF- α			
Non-iners Lactobacillus	–	0.797	***0.0001***
Lactobacillus iners	0.797	–	***0.0001***
Diverse	***0.0001***	***0.0001***	–
C-reactive protein			
Non-iners Lactobacillus	–	***0.004***	***0.0001***
Lactobacillus iners	***0.004***	–	***0.0001***
Diverse	***0.0001***	***0.0001***	–

^1^p-value for t-test with Bonferroni correction; bold indicates statistical significance for α = 0.05.

Sociodemographic and clinical characteristics of the 436 study participants according to vaginal CST category at the early pregnancy enrollment visit (8-14 weeks gestation) shown in **Table 3**. A number of sociodemographic characteristics varied significantly according to CST category, including maternal age (p=0.009), level of education (p=0.0001), marital or cohabitation status (p=0.001). When considering clinical characteristics, parity (prior birth, p=0.044)) status and the use of marijuana (p=0.001) in the month prior to the early pregnancy enrollment visit (8-14 weeks) were significantly different according to CST category, whereas no other clinical characteristics were, including whether the woman had a diagnosis of reproductive or urinary tract infection or whether she was prescribed antibiotics (oral or parenteral) in the month prior to sampling.

**Table 3:** Sociodemographic and Clinical Characteristics of Women According to CST Category.

CHARACTERISTIC	Non-iners *Lactobacillus *(CST I, II, V) N = 71	*Lactobacillusiners *(CST III) N = 151	Diverse (CST IV) N = 214	p-value^1^
Age in years, mean ± sd	26.4 ± 5.4	25.1 ± 4.7	24.4 ± 4.8	***0.009***
Pregnancy insurance				
Low-income Medicaid	14 (20%)	66 (44%)	78 (36%)	***0.0001***
Pregnancy Medicaid	27 (38%)	51 (34%)	105 (49%)	
Private	30 (42%)	34 (23%)	31 (14%)	
Education				
Less than high school	8 (9%)	18 (12%)	43 (20%)	***0.0001***
High school or GED	18 (25%)	59 (39%)	93 (44%)	
Some college	15 (21%)	56 (37%)	56 (26%)	
College graduate	32 (45%)	18 (12%)	22 (10%)	
Not Married or Cohabiting	29 (41%)	65 (43%)	129 (60%)	***0.001***
Obstetrical history				
Prior birth	36 (51%)	93 (62%)	104 (49%)	***0.044***
Prior preterm birth (among those with birth)	6/36 (17%)	20/93 (22%)	23 (22%)	0.779
First prenatal Body Mass Index				
Underweight	1 (1%)	9 (6%)	8 (4%)	0.266
Healthy weight	33 (47%)	60 (40%)	82 (38%)	
Overweight	19 (27%)	27 (18%)	43 (20%)	
Obese	18 (25%)	55 (36%)	81 (38%)	
Substance use in month prior				
Tobacco	5 (7%)	24 (16%)	40 (19%)	0.067
Marijuana	12 (17%)	45 (30%)	86 (40%)	***0.001***
Alcohol	6 (9%)	17 (11%)	11 (5%)	0.106
Antibiotic use in month prior				
Any parenteral or oral	4 (6%)	21 (14%)	32 (15%)	0.119
Diagnoses in month prior				
Bacterial vaginosis	3 (4%)	8 (5%)	15 (7%)	0.736
Chlamydia	2 (3%)	7 (5%)	9 (4%)	0.900
Gonorrhea	0	0	2 (0.9%)	0.659
Trichomonas	0	4 (3%)	1 (0.5%)	0.153
Urinary tract infection	1 (1%)	13 (9%)	17 (8%)	0.123

^1^p-value for t-test (for continuous variables) or Fisher’s exact test or Chi-square test (for categorical variables); bold indicates statistical significance for α = 0.05.

### Birth Outcome According to CST

Of the 436 participants, 401 had a pregnancy that ended in birth: 59 (15%) PTB, with 44 (11%) spontaneous and 15 (4%) medically-indicated; 111 (28%) ETB, with 84 (21%) spontaneous and 27 (7%) medically-indicated; 231 (58%) full term birth. The remaining 35 participants had a pregnancy that ended in spontaneous abortion and were excluded from analysis. Of note, the distribution of pregnancy outcomes among the 38 women whose vaginal samples were excluded due to low library size were not different from those whose samples were included (19/38 [50%] had a full term birth, 11/38 [29%] had ETB, 6/38 [16%] had PTB, and 2/38 had a spontaneous abortion. Similarly, in comparing the library size according to gestational age at birth outcome, there was no significant difference in library size for those with or sPTB *vs.* full term birth (p=0.90; **Supplement Figure 2A**) or sETB *vs.* full term birth (p=0.32; **Supplement Figure 2B**).

The 359 women included in the comparative analyses that follow are those whose pregnancy ended in either sPTB or sETB, with full term birth serving as the comparison group. Among the 44 women in the sPTB group, the range of gestational weeks were 23-1/7 weeks through 36-6/7 weeks (mean gestational weeks of 33-2/7 ± 3-3/7 days) with 11/44 (25%) being very preterm (< 32 weeks), 2/44 (5%) being moderate preterm (32 through 33-6/7 weeks) and 31/44 (70%) being late preterm (34 through 36-6/7 weeks). Among the 84 women in the sETB group, the range of gestational age were 37-0/7 weeks through 38-6/7 weeks, with mean gestational weeks of 37-6/7 ± 3/7 weeks). **Table 4** shows the sociodemographic and clinical characteristics of these participants according to birth outcome. There were significant differences in maternal pregnancy insurance type, parity, body mass index at first prenatal visit, and marijuana use when comparing those with sPTB and sETB to those with full term birth; these variables were controlled for in multivariate analyses examining the relationship between the vaginal microbiome and birth outcome.

**Table 4:** Sociodemographic and Clinical Characteristics of Women According to Birth Outcome.

CHARACTERISTIC	Spontaneous Preterm N = 44	Spontaneous Early Term N = 84	Full Term (ref)^1^ N = 231
Age, mean ± sd	*p=0.721*	*p=0.475*	
	24.9 ± 4.9	25.2 ± 4.9	24.7 ± 4.7
Pregnancy insurance	p=0.022*	*p=0.793*	
Low-income Medicaid	21 (48%)	29 (34%)	82 (36%)
Pregnancy Medicaid	20 (46%)	36 (43%)	90 (39%)
Private	3 (7%)	19 (23%)	59 (26%)
Education	*p=0.167*	*p=0.286*	
Less than high school	8 (18%)	10 (12%)	35 (15%)
High school or GED	21 (48%)	38 (45%)	77 (33%)
Some college	11 (25%)	23 (27%)	76 (33%)
College graduate	4 (9%)	13 (16%)	43 (19%)
Not Married or Cohabiting	*p=0.088*	*p=0.760*	
	32 (64%)	42 (50%)	120 (52%)
Prior birth	*p=0.130*	p=0.026*	
	27 (61%)	53 (63%)	113 (49%)
Body Mass Index	p=0.019*	p=0.047*	
Underweight	1 (2%)	0	11 (5%)
Healthy weight	20 (46%)	40 (48%)	86 (37%)
Overweight	15 (34%)	19 (23%)	44 (19%)
Obese	8 (18%)	25 (30%)	90 (39%)
Substance use in month prior			
	*p=0.522*	*p=0.583*	
Tobacco	6 (14%)	11 (13%)	36 (16%)
	*p=0.601*	***p=0.043***	
Marijuana	14 (32%)	20 (24%)	83 (36%)
	*p=0.318*	*p=0.585*	
Alcohol	2 (5%)	6 (7%)	21 (9%)
Antibiotics in month prior			
Any parenteral or oral	*p=0.473* 4 (9%)	*p=0.712* 10 (12%)	32 (14%)
Diagnosis in month prior			
	*p=0.051*	*p=0.296*	
Bacterial vaginosis	0	4 (5%)	19 (8%)
	*p=0.999*	P=0.999	
Chlamydia	1 (2%)	4 (5%)	10 (4%)
	*p=0.160*		
Gonorrhea	1 (2%)	0	0
	*p=0.999*	p=0.577	
Trichomonas	0	0	4 (1.7%)
	*p=0.999*	P=0.414	
Urinary tract infection	3 (7%)	4 (5%)	17 (7%)

^1^p-value indicates the result of the Chi-square test of significance with full term births as referent category; bold indicates statistical significance for α = 0.05.

**Table 5** shows the proportion of women in the five CST (top portion) and three CST categories (bottom portion) according to the birth outcomes of interest. Because of the low proportion of women with vaginal CST II and V, we collapsed these with CST I to create the non-iners *Lactobacillus* CST category. Likewise, because of the low proportion of women with CST VI-C, and the non-significant differences in the birth outcomes of interest for women with CST IV-A vs. CST IV-B (p=0.840), we combined CST IV-A, IV-B, and IV-C into a single category (Diverse CST).

**Table 5:** Proportion of Women with Vaginal Community State Type According to Birth Outcome.

Vaginal Community State Type	Spontaneous Preterm N = 44	Spontaneous Early Term N = 84	Full Term *(ref)^1^* N = 231
**CST**	*p=0.068*	*p=0.716*	
CST I *(L. crispatus)*	1 (2%)	14 (17%)	25 (11%)
CST II *(L. gasseri)*	0	1 (1%)	6 (3%)
CST V *(L. jenseni)*	0	4 (5%)	9 (4%)
CST III *(L. iners)*	12 (27%)	26 (31%)	86 (37%)
CST IV-A (Diverse-A)	17 (39%)	21 (25%)	58 (25%)
CST IV-B (Diverse-B)	14 (32%)	18 (21%)	44 (19%)
CST IV-C (Diverse-C)	0	0	3 (1%)
**CST Category**	p=0.004**	*p=0.445*	
CST I, II, V (Non-iners *Lactobacillus*)	1 (2%)	10 (23%)	40 (17%)
CST III *(Lactobacillus iners)*	12 (27%)	26 (31%)	86 (37%)
CST IV-A, IV-B, IV-C (Diverse)	31 (71%)	39 (46%)	105 (45%)

^1^p-value indicates the result of the Chi-square test of significance with full term births as referent category; bold indicates statistical significance for α = 0.05.

**Figure 3:**
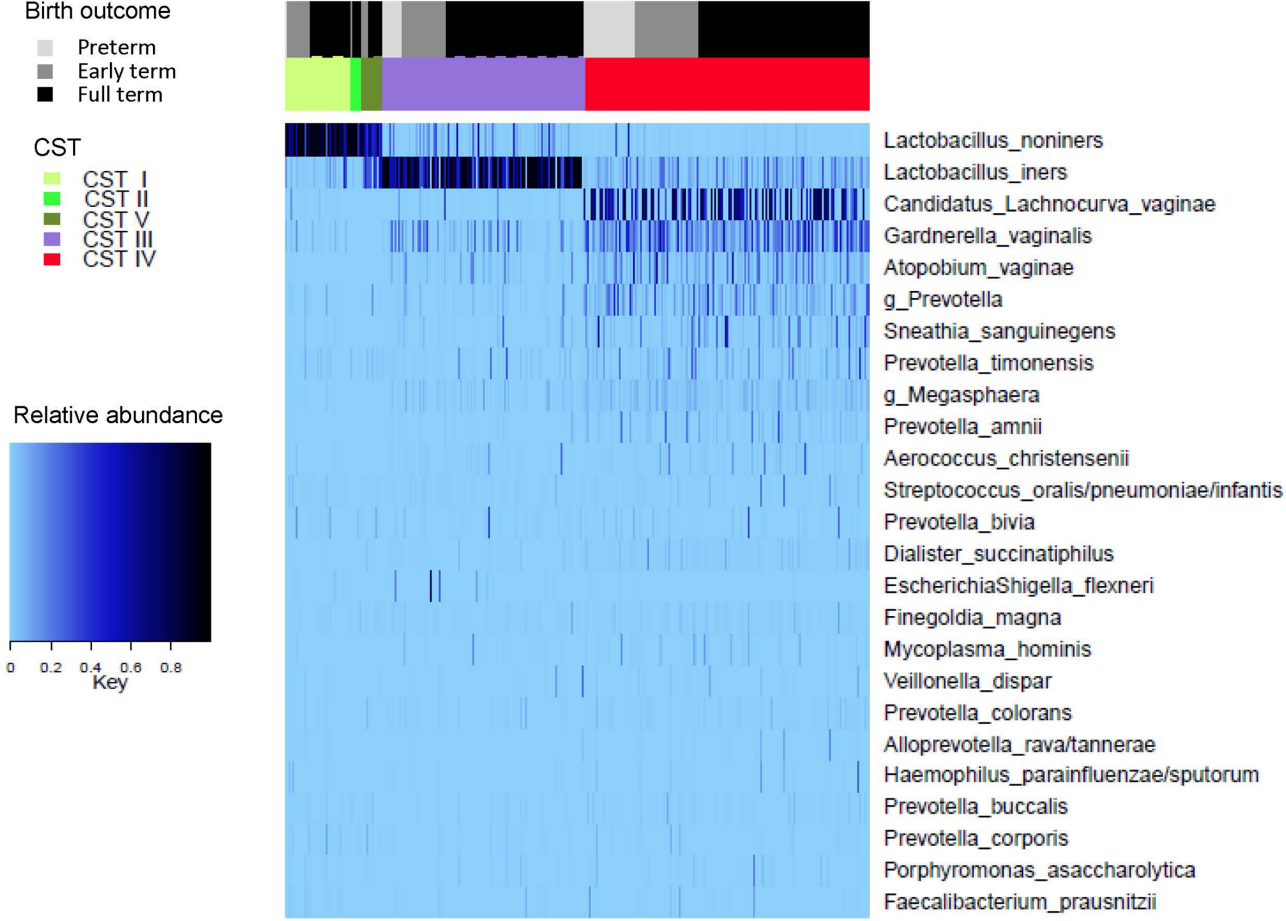
Top 25 taxa, grouped according to Community State Type (CST) and Gestational age at birth outcome.

A heat map representing the composition of the vaginal microbiome, as classified by CST, according to gestational age at birth outcome is given in **Figure 3**.

**Table 6** shows the results of multivariate logistic regression modeling, controlling for key co-variates that associate with gestational age at birth outcomes among our cohort or in the established literature. The upper portion of **Table 6** shows the Diverse CST (Diverse-A, Diverse-B, and Diverse-C combined) whereas the lower portion shows the Diverse CST subcategories. Compared to women in the non-iners *Lactobacillus* CST, those in the Diverse CST had substantially and significantly elevated adjusted odds of sPTB (aOR = 7.7 [1.8, 72.0]) but not sETB (aOR = 0.9 [0.4, 1.8]). Within the Diverse CST, both Diverse-A and Diverse-B were strongly significantly associated with sPTB with aOR = 7.6 (1.7, 73.1) and 8.2 (1.8, 79.3), respectively, whereas Diverse-C was not (aOR = 3.5 [0.01, 108.5]), acknowledging a very small sample size for the Diverse-C CST. The two-sided confidence intervals for the adjusted odds of sPTB or sETB for those in the *Lactobacillus iners* CST (CST III) compared to the non-iners *Lactobacillus* CST included the null value of 1. However, as the upper confidence limits for the odds of sPTB were very large and appeared to support a directional hypothesis, we also present one-sided 95% confidence intervals that recognize the upper limits of the two-sided intervals are essentially infinite (Ruxton and Neuhäuser, 2010). The one-sided confidence interval for the *Lactobacillus iners* CST excludes the null value of 1 (aOR=4.0 [1.1, inf]), thus showing significantly increased adjusted odds of sPTB relative to the non-iners *Lactobacillus* CST.

**Table 6:** Odds of Birth Outcome According to CST (5-Category) *(Full Term = Referent)*.

Vaginal CST Category	OR (95% Confidence Interval)			
	Spontaneous Preterm N = 44		Spontaneous Early Term N = 84	
	**unadjusted**	**adjusted** *^1^*	**unadjusted**	**adjusted** *^1^*
Non-iners Lactobacillus	*Ref*	*Ref*	*Ref*	*Ref*
Lactobacillus iners	3.9 (0.89, 36.7) **(1.1, inf)^#^**	4.0 (0.8, 38) **(1.1, inf)^#^**	0.6 (0.3, 1.3)	0.6 (0.3, 1.2)
Diverse	**8.1 (2.0, 73.4)** **(2.4, inf)** ^#^	**7.7 (1.8, 72.0)** **(2.2, inf)** ^#^	0.8 (0.4, 1.5)	0.9 (0.4, 1.8)
Diverse-A	**8.1 (1.9, 75.1)** **(2.3, inf)** ^#^	**7.6 (1.7, 73.1)** **(2.1, inf)** ^#^	0.8 (0.4, 1.6)	0.8 (0.4, 1.9)
Diverse-B	**8.8 (2.0, 82.7)** **(2.5, inf)** ^#^	**8.2 (1.8, 79.3)** **(2.2, inf)** ^#^	0.9 (0.4, 1.9)	1.0 (0.4, 2.3)
Diverse-C	3.9 (0.02, 89.1)	3.5 (0.02, 108.5)	0.3 (0.002, 3.3)	0.3 (0.002, 4.0)

^1^Co-variates included in the multivariate model include age, education level, insurance type, marital-cohabitation status, parity, first prenatal BMI category, tobacco use, marijuana use, gestational age of sample.

^#^One-sided confidence interval; bold indicates statistical significance for α = 0.05.

### Birth Outcome According to Relative Abundance and Presence/Absence of Taxa

When applying the LDM to those with sPTB and full term birth (275 samples), there were 324 ASVs that remained after filtering out ASVs present in less than 5 samples. In the LDM that considered relative abundance, the global p-value for the effect of the vaginal microbiome on the outcome of sPTB was statistically significant (global p-value = 0.034), yet no ASVs were significant after controlling for multiple comparisons at a FDR of 10%. The top 10 smallest p-values (q-values ≥ 0.348) yielded by the LDM that considered relative abundance corresponded to a higher relative abundance of *Paraprevotella clara* (p=0.001), *Collinsella aerofaciens* (p=0.006), *Ruminococcus callidus* (p=0.008), *Gardnerella vaginalis* (p=0.011), *Paraeggerthella hongkongensis* (p=0.014), an ASV that classified to genus *Prevotella* (p=0.016), *Intestinibacter barlettii* (p=0.017), and *Alistipes putredinis* (p=0.019) and *Catenibacterium mituokai* (p=0.018) and a lower relative abundance of non-iners *Lactobacillus* (p=0.016). In this LDM that considered relative abundance, none of the 19 pre-specified taxa achieved significance when accounting for multiple comparisons (q-values ≥ 0.498) but the relative abundance of three taxa, namely, the higher relative abundance of *Gardnerella vaginalis* and *Mobiluncus curtisii* and the lower relative abundance of non-iners *Lactobacillus*, had significant raw p-values (**Table 7**) using the nominal 5% cutoff. Of note, in sensitivity analyses, we compared the relative abundance of dropped taxa for those whose pregnancy ended in full term birth vs. sPTB, and found no significant difference (p=0.15; **Supplement Figure 3**), supporting that the removal of taxa from analysis did not influence our findings. In addition, we re-ran the LDM both with the inclusion of read count as a co-variate and found no change in the global p-values as well as with the dropped taxa included and none were identified as significantly different according to gestational age at birth outcomes.

**Table 7:** Raw p-values for Pre-specific Taxa in the LDM Model^1^ for Spontaneous Preterm Birth and Spontaneous Early Term Birth.

Taxon	Spontaneous Preterm N = 44		Spontaneous Early Term N = 84	
	Relative Abundance Model	Presence/Absence Model	Relative Abundance Model	Presence/Absence Model
*Aerococcus christensenii*	0.170	0.291	0.719	0.195
*Atopobium vaginae*	0.211	***0.049***	0.810	0.137
*BVAB1*	0.209	0.091	0.697	0.062
*BVAB2*	0.077	***0.024***	0.903	0.622
*Dialister microaerophilis*	0.093	***0.011***	0.167	0.271
*Finegoldia magna*	0.924	0.629	0.970	0.981
*Gardnerella vaginalis*	***0.011***	0.382	0.132	0.530
*non-iners Lactobacillus*	***0.016***	0.307	0.654	0.449
*Lactobacillus iners*	0.308	0.834	0.123	0.318
*g_Megasphaera*	0.345	0.436	0.052	0.303
*Mobiluncus curtisii*	***0.035***	0.244	0.346	0.280
*Mycoplasma hominis*	0.737	0.423	0.274	0.141
*Prevotella amnii*	0.296	***0.044***	0.934	0.188
*Prevotella bivia*	0.848	0.517	0.906	0.915
*Prevotella buccalis*	0.391	0.498	0.053	0.443
*Prevotella timonensis*	0.722	0.853	0.849	0.689
*Ureaplasma urealyticum*	0.624	0.673	0.440	0.080
*Sneathia amni*	0.782	0.952	0.127	0.203
*Sneathia sanguinegens*	0.511	0.390	0.138	0.961

^1^Co-variates included in the multivariate model include age, education level, insurance type, marital-cohabitation status, parity, first prenatal BMI category, tobacco use, marijuana use, gestational age of sample; bold indicates statistical significance for α = 0.05.

In the LDM that considered the presence/absence of ASV on the outcome of sPTB, the global p-value was 0.328. In this presence/absence LDM model, no ASVs were significantly associated with sPTB after controlling for multiple comparisons. The top 10 smallest p-values (q-values ≥ 0.661) yielded by the LDM model corresponded to the presence of these ASVs: *Dialister succinatiphilus* (p=0.002), *Paraprevotella clara* (p=0.006), *Dialister microaerophilis* (p=0.011), *Bifidobacterium breve* (p=0.011), *Paraeggerthella hongkongensis* (p=0.014), *Intestinibacter barlettii* (p=0.015), *Roseburia inolinivorans* (p=0.020), BVAB2 p= (0.024), *Atopobium deltae* (0.031), and *Ruminococcus callidus* (p=0.0345). In the LDM model that considered presence/absence, none of the 19 pre-specified taxa achieved significance when accounting for multiple comparisons (q-values ≥ 0.818) although the presence of four taxa, namely, *Atopobium vaginae*, BVAB2, *Dialister microaerophilis*, and *Prevotella amnii*, had significant raw p-values (**Table 7**) using the nominal 5% cutoff.

When applying the LDM to the sample that included sETB and full term birth (315 samples), we used the same 324 ASVs as the sPTB analysis. In the LDM that considered relative abundance, the global p-value for the effect of the vaginal microbiome on sETB was not significant (global p-value = 0.320), however, the higher relative abundance of seven ASVs were associated with sETB with FDR < 10% (q-value 0.0299-0.0471), including *Dialister invisus* (p=0.00016), *Blautia luti* (p=0.0002), *Collinsella aerofaciens* (p=0.0003), *Campylobacter hominis* (p=0.00042), *Bifobacterium kashiwanohense* (p=0.0005), *Bacteroides vulgatus* (p=0.001), *Bacteroides xylanisolvens* (p=0.0009). In the LDM that considered relative abundance, none of the 19 pre-specified taxa achieved significance when accounting for multiple comparisons (q-values ≥ 0.414) although two taxa had borderline significant raw p-values (**Table 7**) using the nominal 5% cutoff: *Prevotella buccalis* (p=0.053) and an ASV belonging to genus *Megasphaera* (p=0.052).

In the LDM that considered the presence/absence of ASV on the outcome of sETB, the global p-value was significant (p=0.030) and the presence of 13 ASVs were associated with sETB with FDR < 10% (q-value 0.017 – 0.084), including *Bifidobacterium breve* (p<0.0001), *Blautia luti* (p=0.0002), *Bifidobacterium longum* (p=0.0004), *Akkermansi muciniphila* (p=0.0005), *Citrobacter freundi/gillenii/youngae* (p=0.0006), *Bifidobacterium kashiwanohense* (p=0.0007), *Dialister invisus* (p=0.0014), *Klebsiella pneumonia/variicola/oxytoca* (p=0.0023), *Veillonella rogosae* (p=0.0037), *Parabacteroides distasonis* (p=0.0030), *Bacteroides xylanisolvens* (p=0.0028), *Collinsella aerofaciens* (p=0.003), *Alistipes putredinis* (p=0.0033). In the LDM that considered presence/absence, none of the 19 pre-specified taxa achieved significance when accounting for multiple comparisons (q-values ≥ 0.475) and none had a significant raw p-value (**Table 7**).

**Figure 4:**
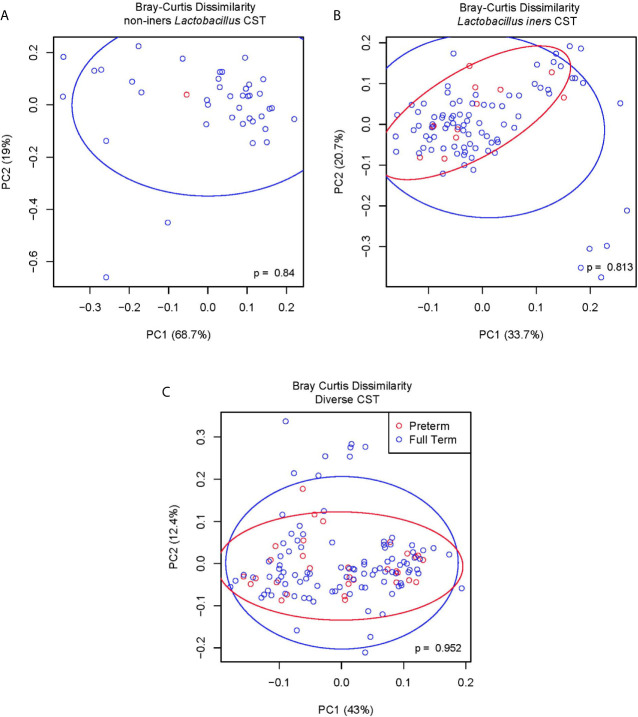
**(A)** Bray-Curtis dissimilarity non-iners *Lactobacillus* CST. **(B)** Bray-Curtis dissimilarity *Lactobacillus iners* CST. **(C)** Bray Curtis dissimilarity diverse CST.

In order to discern whether particular ASV within the Diverse CST (CST IV) were associated with the occurrence of sPTB, we applied the LDM model to only those study participants with Diverse CST considering 11 taxa comprised of the 9 most abundant ASV within CST IV (BVAB1, *Garderella vaginalis, Atopbium vaginae*, an ASV in genus *Prevotella, Sneathia sanguinegens, Prevotella timonensis, Prevotella amnii, Aerococcus christensenii*, an ASV in genus *Megasphaera*) in addition to *L. iners* and non-iners *Lactobacillu*s. For the relative abundance LDM for sPTB vs. full term birth (N = 136 samples) within the Diverse CST, the global p-value for the vaginal microbiome was not significant (p=0.888) and the relative abundance of none of these 11 ASV were associated with sPTB when accounting for multiple comparisons, although the raw p-value for one ASV was nominally significant (*Aerococcus christensenii*, p=0.0367) at the 5% level. For the presence/absence modeling for sPTB vs. full term birth within the Diverse CST, the global p-value also was not significant (p=0.448) and the presence/absence of none of the 11 ASVs were associated with sPTB in accounting for multiple comparisons although the raw p-value for one ASV was nominally significant (*Atopobium vaginae*, p=0.0392) at the 5% level. To further investigate whether the differential relative abundance of particular taxa within each CST category associated with sPTB we calculated the Bray-Curtis dissimilarity index for those with sPTB vs. full term birth; for each CST category, we show (**Figure 4**) that the within CST category composition did not significantly differ based on sPTB vs. full term birth status (non-iners *Lactobacillus* CST, p=0.84; *Lactobacillus iners* CST, p=0.81; Diverse CST, p=0.95).

## Discussion

This study adds to the growing literature characterizing the vaginal microbiome and sPTB among AA women, who have been underrepresented in research despite having higher rates of PTB relative to US women of other races/ethnicities. In this cohort of AA women, those with an early pregnancy vaginal CST IV (Diverse CST) had a substantially and significantly elevated risk for sPTB compared to AA women with a vaginal microbiome classified as CST I, II, or V (non-iners *Lactobacillus* dominated), and those with vaginal CST III (*L. iners* dominated) were of intermediate risk between CST IV and CST I, II, or V (non-iners *Lactobacillus* dominated). Notably, nearly half (49%) of women in the Atlanta African American cohort had an early pregnancy vaginal microbiome classified as CST IV (Diverse CST) while nearly one-third (35%) had CST III (*L. iners* dominated). Despite finding a strong association between vaginal CST IV and risk of sPTB in this AA cohort, neither the relative abundance nor presence/absence of any particular ASV was associated with sPTB when considering women across all CST categories or within CST IV. In particular, within the three CST groups, we found no evidence of differences in microbial composition between women who experienced sPTB and women who delivered full term, further suggesting that these CST groupings are sufficient to explain the patterns we observed.

In this cohort of AA women, there was no significant association between vaginal CST in early pregnancy and risk of sETB, however, the LDM identified 7 ASV (*Bacteroides vulgatus, Bacteroides xylanisolvens, Bifidobacterium kashiwanohense, Blautia luti, Campylobacter hominis, Collinsella aerofaciens Dialister invisus*) whose higher relative abundance and 13 ASV (*Akkermansi muciniphila, Alistipes putredinis, Bacteroides xylanisolvens, Bifidobacterium breve, Bifidobacterium kashiwanohense, Bifidobacterium longum, Blautia luti, Citrobacter freundi/gillenii/youngae, Collinsella aerofaciens, Dialister invisus, Klebsiella pneumonia/variicola/oxytoca, Parabacteroides distasonis, Veillonella rogosae*) whose presence was associated with sETB. Notably, these ASV identified in both the relative abundance and presence/absence model are those typically considered as gut-associated, although they are not the most common taxa found in fecal samples (Truong et al., 2017). Previous research has documented the presence of gut and oral bacterial populations in the vaginal microbiome, particularly in cases of bacterial vaginosis, and has suggested that their presence may be linked to fecal or oral transplantation (Africa et al., 2014; Fenollar and Raoult, 2016). Previous research has described *Bifidobacterium breve* and *B. longum* occurring as dominant members of the vaginal microbiome (Freitas and Hill, 2017) and that the vagina and gut are, in fact, colonized by a shared community of *Bifidobacterium* (Freitas and Hill, 2018). To rule out the possibility that these “gut-like” taxa that we observed as associated with sETB were artifactual, we conducted sensitivity analyses. First, we repeated our LDM analyses after removing data from three women with sETB that had the highest abundance of these “gut-like” taxa. Second, based on counts for these taxa observed in our no-template controls, we applied a threshold to require all ASVs to have a raw count of 20 or more to be included in the LDM analyses, which were repeated using both relative abundance and presence-absence data. In all of these instances, the conclusions were essentially unchanged and the taxa with the smallest raw p-values continued to be these “gut-like” taxa. Finally, we note that case samples (sPTB and sETB) were carefully balanced across extraction batches, PCR plates and sequencing lanes in these experiments.

In summary, the findings from this study support that the early pregnancy microbiome of AA women is important in the risk of both sPTB and sETB. In this cohort of AA women, an early pregnancy vaginal CST III or IV was associated with an increased risk of sPTB but not an increased risk of sETB. The LDM based on relative abundance found a significant overall effect of the vaginal microbiome on sPTB (p=0.034) but not sETB (p=0.320), whereas the LDM based on presence/absence of ASV found no overall effect on sPTB (p=0.328) but a significant effect on sETB (p=0.030). Using the LDM to test for ASV-specific effects, no ASV was significantly associated with sPTB considering either relative abundance or presence/absence data after controlling for multiple comparisons (FDR 10%), although in marginal analyses of taxa previously shown to be associated with sPTB, the relative abundance of *Gardnerella vaginalis* (p=0.011), non-iners *Lactobacillus* (p=0.016), and *Mobiluncus curtisii* (p=0.035) and the presence of *Atopobium vaginae* (p=0.049), BVAB2 (p=0.024), *Dialister microaerophilis* (p=0.011), and *Prevotella amnii* (p=0.044) were associated with sPTB. The LDM identified the higher abundance of 7 ASV and the presence of 13 ASV, all commonly residents of the gut, as associated with sETB at FDR of 10%. Although the clinical literature conveys that the occurrence and recurrence of PTB and ETB share common risk factors that suggests that these outcomes share a common etiology and reflect a continuum of risk related to shortened gestation (Delnord and Zeitlin, 2019), our findings support that the microbial risks may be unique. It is difficult to speculate as to why the presence of gut-associated bacteria would be associated with sETB but not sPTB, but this may relate to differences in inflammatory milieu or microbial products of the gut-like taxa. We plan to explore this area of research further through a metagenomics approach that allows for functional profiling of these bacterial taxa and their virulence properties. Taken together, these findings suggest that it is the overall composition of the vaginal microbiome with respect to common taxa, particularly the low relative abundance of non-iners *Lactobacillus* spp. that is the relevant feature in shaping sPTB risk, whereas it is the relative abundance or presence/absence of rare, potentially pathogenic taxa that is relevant in shaping sETB risk. An alternative explanation for these findings, however, is that the quantitative level (qPCR concentrations) of minority and pathogenic bacterial taxa may be a driver of sPTB, but exploration of this issue is beyond the scope of this project.

To our knowledge, this is the first study to report on the association between the vaginal microbiome and risk of sETB. Our findings around the association between the vaginal microbiome and risk of sETB are important for contextualizing and interpreting earlier vaginal microbiome and birth outcome studies, many of which did not distinguish ETB as a category but rather included births in this category in the “term birth” category. While more research in a broader study population is warranted, our findings support that the inclusion of ETB in the comparison category of term births could result in bias.

Direct comparison of the findings of our study with those of other studies of the vaginal microbiome and PTB is difficult as there is substantial heterogeneity in methods across published studies, and differing methods of DNA extraction, PCR amplification (including the selection of particular primers), bioinformatic processing and taxonomic classification are known to impact the sensitivity for identifying particular taxa (Stackebrandt and Goebel, 1994; Gill et al., 2016; Gohl et al., 2016). However, it is notable that our findings around the importance of the relative abundance of non-iners *Lactobacillus* and *L. iners* are similar to those reported previously for an AA cohort, which found that a lower abundance of *L. crispatus*, *L. gasseri*, and *L. jensenii* were significantly associated with PTB risk. This agreement is more remarkable as that cohort was restricted to women with a prior history of sPTB who received progesterone therapy in the subsequent pregnancies that comprise their cohort (Callahan et al., 2017b). Also, in a study restricted to women with a prior sPTB, vaginal CST IV was found to be a significant risk factor for a recurrent sPTB (Gerson et al., 2020). Conversely, studies that have considered substantial numbers of cases of PTB among AA women did find particular taxa that increased the risk of PTB, whereas our study did not. Specifically, in a cohort focused on AA women (45 with sPTB, 90 with term birth matched for age and income) those with sPTB had significantly lower abundance o*f L. crispatus*, higher abundance of BVAB1 (candidate name *Candidatus Lachnocurva vaginae*), *Sneathia amnii*, TM7-HI, and a group of Prevotella whereas women that delivered term were more likely to have *L. crispatus* and decreased prevalence of *A. vaginae* and *G. vaginalis* (Fettweis et al., 2019). In another cohort of predominantly AA women (107 with sPTB, 432 with term birth), seven specific taxa were significantly associated with increased risk of sPTB (*Sneathia sanguinegens, Mobiluncus curtisii/mulleris, Mageeibacillus indolicus, Megasphaera, Porphyromonas asaccaraolycia, Prevotella buccalis, Atopbium*) with a stronger effect among AA women (Elovitz et al., 2019). Also, in contrast to our study’s findings, that same study found an association between CST IV and sPTB among non-AA women but no significant association among AA women (Elovitz et al., 2019). Another predominantly AA cohort study also found no association between vaginal CST and sPTB vs. term birth (Romero et al., 2014a). It is possible that differences in the timing of sampling collection (8-14 weeks gestation for our study in contrast to 24 weeks or fewer for the other studies) contribute to differences in findings among AA women, given that the vaginal microbiome composition does change across pregnancy (Romero et al., 2014a; Romero et al., 2014b). The significance of vaginal CST IV in early pregnancy upon sPTB risk suggests that the vaginal composition early in pregnancy is more influential upon PTB risk than that in mid-pregnancy.

Findings from this study also supports that the CST IV (Diverse CST) is associated with a vaginal cytokine profile that is more pro-inflammatory than that of CST I, II, or V (non-iners *Lactobacillus*) or CST III (*L. iners*), which is consistent with other research. Vaginal lactobacilli, including *L. crispatus*, *L. gasseri*, and *L. jensenii*, are known to play a critical role in regulating the inflammatory response in the female genitourinary tract (Anahtar et al., 2015), with *L. iners* having a comparatively upregulated inflammatory response (Doerflinger et al., 2014). A study that contrasted the inflammatory profile of a vaginal microbiome dominated by *L. crispatus* vs. a vaginal microbiome dominated by *A. vaginae* found unique species-specific innate immune signatures with *L. crispatus* colonization resulting in low epithelial cell activation and minimal disruption of the immune barrier properties and *A. vaginae* inducing a robust inflammatory profile that disrupts physiochemical barrier properties of the vaginal mucosa (Doerflinger et al., 2014). Experimental research also supports that cytokine production by vaginal epithelial cells in response to stimuli varies according to vaginal microbiome composition. Vaginal epithelial cells harvested from women with a vaginal microbiome not dominated by *Lactobacillus* have been found to produce less lactic acid and induce greater inflammatory cytokine production of IL-1α, IL-1β, IL-6, IL-8, macrophage inflammatory protein (MIP)-1α, and MIP-1β in response to the presence of *Gardnerella* relative to epithelial cells from women whose vaginal microbiome is *Lactobacillus* dominated (Manhanzva et al., 2020). The prevailing theory is that, when *Lactobacillus* dominance is lost and vaginal microbial diversity increases, the production of pro-inflammatory cytokines along with the recruitment of immune cells and reduced viscosity of the cervicovaginal fluid result in changes in immune and epithelial homeostasis, which ultimately affect the barrier properties of the genital epithelium and cervix, increasing the risk for ascending infection (Amabebe and Anumba, 2018; Torcia, 2019). Ascending intrauterine infection early in gestation accounts for an estimated 50% of spontaneous PTB (Goldenberg et al., 2008).

This study has substantial strengths for investigating the role of vaginal microbiome composition and PTB among AA women. First, this study exclusively focused on AA women and, as such, had a relatively large number of cases of PTB across CST categories to consider the within-race risk of PTB according to CST. Second, our protocol allowed for the careful phenotypic differentiation of PTB into sPTB and medically-indicated categories based on in-depth review of the prenatal and labor and delivery record, minimizing the opportunity for misclassification which might affect studies of self-report of type of PTB. Although a related issue is that this study restricted enrollment to women without chronic medical conditions, in order to reduce the number of early deliveries that were attributable to medical indications, which may affect the generalizability of our findings to the broader population of African American women who give birth, a substantial proportion of whom would be expected to have chronic medical conditions (DeSisto et al., 2018; Harris et al., 2020). Third, in this study we also controlled for known socioeconomic and clinical risk factors for PTB (as identified in the literature and as occurring among our study population) as well as socioeconomic factors associated with CST in our study population (such as maternal education and health insurance status), strengthening the likelihood that the association between CST IV and PTB is a true association rather than confounding. Finally, the vaginal samples analyzed here were collected within a fairly narrow gestational age window during early pregnancy.

This study is not without limitations, most of which have affected other existing studies of the microbiome. The potential to discover microorganisms whose presence in the vaginal tract is related to the occurrence of sPTB is limited by the inherent low resolution of 16S amplicon sequencing. Despite the known polymicrobial nature of PTB (Payne and Bayatibojakhi, 2014), 16S rRNA gene sequencing cannot identify non-bacterial microbes, such as viruses, fungi, or protozoa, all of which have been linked to PTB risk (Payne and Bayatibojakhi, 2014). Furthermore, 16S rRNA gene approaches are limited in their ability to reliably assign many bacterial genera to the species level and to distinguish strains (Frank et al., 2008; Fettweis et al., 2012; Poretsky et al., 2014). Strains within a species have variability in capacity for virulence due to differences in accessory genes (Tett et al., 2017). Within the vaginal microbiome, the presence of a few highly dominant species common among women experiencing either PTB or term birth (e.g., *L. iners, G. vaginalis*) suggests that crucial differences in the microbiome occur at the *strain level*. Emerging evidence supports that strain-level differences may contribute to PTB risk including that the genome of some *L. iners* strains encode inerolysin, a pore-forming toxin related to vaginolysin of *G. vaginalis*, suggesting clonal variants that in some cases promote a healthy vagina and in others dysbiosis and disease (Petrova et al., 2016); a strain of *G. vaginalis* explains the genus association with PTB in some but not other studies (Callahan et al., 2017a); a strain of *Sneathia amnii* may confer virulence features to BV-associated bacteria and may explain the variable association of BV and the genus *Sneathia* with PTB (Harwich et al., 2012).

In future analyses, we will focus on exploring particular social, environmental, and biobehavioral exposures that may contribute to the high prevalence of CST IV among women in the Atlanta African American cohort as well as employing whole-genome shotgun sequencing to better understand the potential role of particular bacterial strains – especially those present within the CST IV – whose accessory genes might drive risk for sPTB. An understanding of associated exposure and risk factors linked to CST IV in early pregnancy, as well as particular strains within CST IV that are linked with sPTB, may identify modifiable risks that could be targeted through health education and/or public health or clinical interventions.

## Data Availability Statement

The original contributions presented in the study are publicly available. This data can be found here: https://www.ncbi.nlm.nih.gov/sra, PRJNA725416.

## Ethics Statement

The studies involving human participants were reviewed and approved by Emory University Institutional Review Board. The patients/participants provided their written informed consent to participate in this study.

## Author Contributions

AD and EC conceived the study design, oversaw data collection, and conceptualized and wrote the manuscript. GS advised on methods, supervised the statistical findings, and contributed to the writing of the manuscript. Y-JH performed the statistical modeling and computations. TR and BP contributed to the design of the study and to the manuscript. MW contributed to the interpretation of results and to the manuscript. AK and AS contributed to the conduct of the experiments and to the manuscript. All authors discussed results, provided critical feedback and helped shape the research, analysis, and manuscript. All authors contributed to the article and approved the submitted version.

## Funding

This study was supported in part by the National Institutes of Health, National Institute of Nursing Research [R01NR014800, K01NR017903], National Institute on Minority Health and Health Disparities [R01MD009064], National Institute of Environmental Health [R24ES029490], the Office of the Director [UH3OD023318], and the Building Interdisciplinary Research Careers in Women’s Health [K12HD085850]. This study was also supported in part by the Emory Integrated Genomics Core, which is subsidized by the Emory University School of Medicine and is one of the Emory Integrated Core Facilities. Additional support was provided by the National Center for Advancing Translational Sciences of the National Institutes of Health under Award Numbers UL1TR000424, UL1TR000454, and UL1TR002378.

## Conflict of Interest

The authors declare that the research was conducted in the absence of any commercial or financial relationships that could be construed as a potential conflict of interest.
