# Supplementary figures and images for: Vaginal Microbiome Composition in Early Pregnancy and Risk of Spontaneous Preterm and Early Term Birth Among African American Women
Source: Front Cell Infect Microbiol. 2021 Apr 29;11:641005. doi: 10.3389/fcimb.2021.641005 (PMC8117784; doi:10.3389/fcimb.2021.641005)

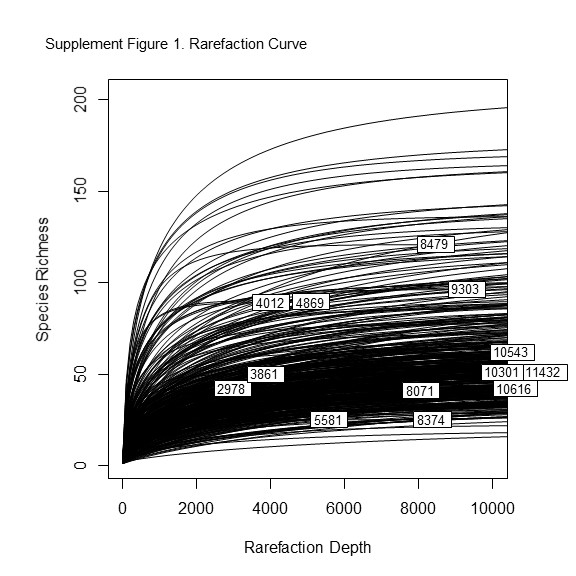

Supplement: Supplementary file 2 [file Image_1.jpeg]

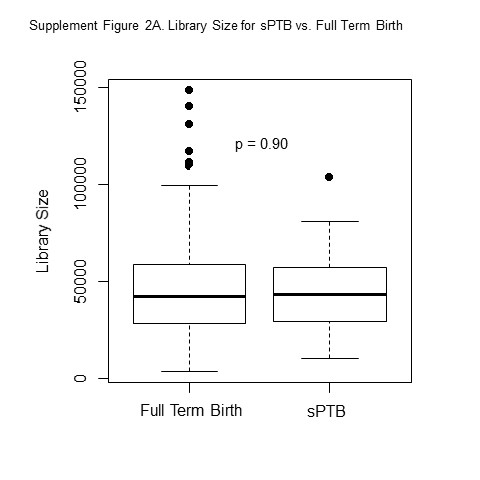

Supplement: Supplementary file 3 [file Image_2.jpeg]

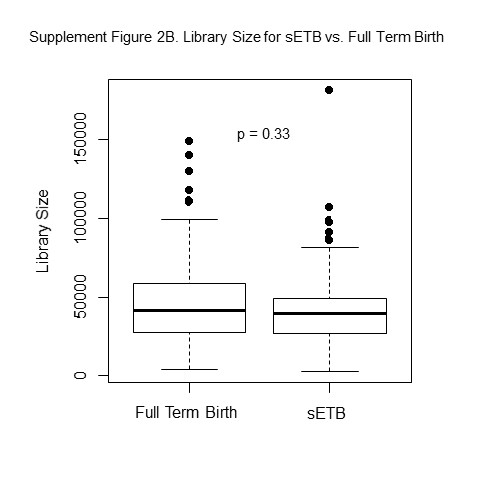

Supplement: Supplementary file 4 [file Image_3.jpeg]

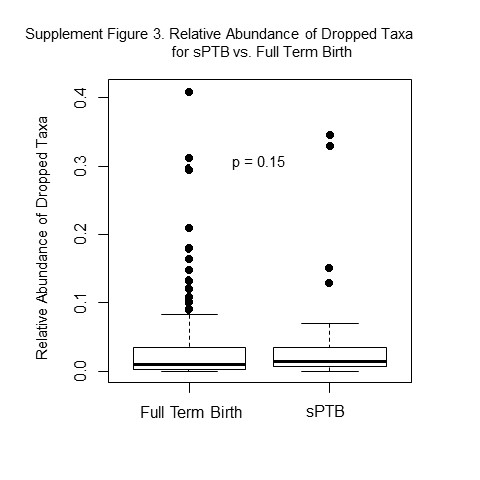

Supplement: Supplementary file 5 [file Image_4.jpeg]
